# Preliminary development of proxy-rated quality-of-life scales for children and adults with Niemann-Pick type C

**DOI:** 10.1007/s11136-019-02234-5

**Published:** 2019-06-21

**Authors:** Lydia Aston, Rachel Shaw, Rebecca Knibb

**Affiliations:** Department of Psychology, School of Life and Health Sciences, Aston University, Aston Triangle, Birmingham, B4 7ET UK

**Keywords:** Lifeworld theory, Niemann-Pick type C, Phenomenology, Proxy scales, Quality of life

## Abstract

**Objectives:**

Niemann-Pick disease type C (NPC) is a rare life-limiting disease for which there is no cure. No scales currently exist to measure the impact of medication, physical therapy or clinical trials. The aim of this study was to develop age-appropriate Quality-of-Life (QoL) scales to measure the impact of NPC on children and adults.

**Design:**

Scale development study using a phenomenological approach to data generation and analysis.

**Methods:**

Fourteen interviews were conducted with people living with NPC and/or their parents/carers. Themes were generated and examined against an existential-phenomenological theory of wellbeing. A matrix was constructed to represent the phenomenological insight gained on participants’ subjective experiences and a bank of items that were related to their QoL was developed.

**Results:**

NPC quality-of-life questionnaires for children (NPCQLQ-C) and adults (NPCQLQ-A) proxy prototype scales were produced and completed by 23 parents/carers of children (child age mean = 8.61 years) and 20 parents/carers of adults (adult age = 33.4 years). Reliability analysis resulted in a 15-item NPCQLQ-C and a 30-item NPCQLQ-A, which showed excellent internal consistency, Cronbach’s *α* = 0.925 and 0.947, respectively.

**Conclusion:**

The NPCQLQ-C and NPCQLQ-A are the first disease-specific QoL scales to be developed for people living with NPC. This novel approach to scale development values the experiential, real life impact of living with NPC and focused on the lived-experiences and impact on QoL. The scales will enable healthcare professionals and researchers to have a better understanding and quantifiable measurement of the impact of living with NPC on a patient’s daily life.

## Introduction

Niemann-Pick disease type C (NPC) is a rare, genetic neurovisceral disease with a clinical incidence of approximately 1:100,000 [1, 2]. The clinical features of NPC include progressive loss of vision, hearing, muscle co-ordination and mobility. Cognitive decline leading to early-onset dementia can occur resulting in premature death in most people [2]. Symptoms are heterogeneous and can arise at any point between early childhood and late adulthood and progress at different rates [1]. NPC is classed as a life-limiting condition as there is no cure, and the only approved disease-specific drug available is Miglustat (Zavesca^**®**^; Actelion Pharmaceuticals Ltd), which is offered for the treatment of progressive neurological manifestations [2].

Living with any life limiting disease brings difficulties but living with a rare disease generates unique challenges for both the person diagnosed and their carers, and can be an isolating experience [3]. Bury’s [4] concept of biographical disruption suggested that an individual’s sense of self, social interactions, and daily life are disrupted by the illness experience. Illness can be characterised as a disturbance to the narrative flow of individuals’ lives, and episodes of ill health can represent significant change and can be viewed as a threat to this constructed self-narrative [5]. When related to NPC, a disruption may feel permanent due to there being no cure, never allowing a return to life prior to the diagnosis; thus necessitating a change in a person’s biography and self-concept [4]. Yet, Carel [6] argued that it was possible for one to experience wellbeing in spite of illness. Thus, living with illness does not necessarily mean a wholly negative disruption and instead could be seen as something that could bring positive consequences, self-discovery and self-development [7, 8]. This illustrates the complexity of the illness journey and that there are numerous factors to take into account when considering the impact living with NPC has on a person’s QoL.

The concept of QoL is a highly valued focus of research and consideration within clinical practice. Despite this popularity, the lack of consensus on the definition of QoL is a source of confusion [9]. Arguably one of the most respected definitions of QoL is the World Health Organisation’s (WHO) which describes it as being “individuals’ perceptions of their position in life in the context of the culture and value systems in which they live and in relation to their goals, expectations, standards and concerns” (p. 1404) [10]. It is therefore multi-dimensional in its outworking and is understood as a “broad ranging concept affected in a complex way by the person’s physical health, psychological state, level of independence, social relationships, personal beliefs and their relationship to salient features of their environment” (p. 1) [11]. With no qualitative research being conducted on understanding the experiences of living with NPC, it was important to prioritise focus on the “individuals’ perceptions” of what life with NPC means, in order to garner the nuances of the illness experience.

Accomplishing this necessitates an engagement with broader philosophical constructs and frameworks that can be used to understand such phenomena. An interaction with other theories that seek to broaden the scope of enquiry and ask more fundamental questions about how human beings understand the world about them is needed. There is a need to step back from the traditional fragmented components of what makes QoL, and subsequently what are used in QoL measurements and instead start first by considering the depth to what constitutes life in all of its quality from an ontological perspective, an issue deeply embedded in what it means to be human. Arguably a stronger emphasis on the developmental process may provide a more robust basis for QoL scales based on lived-experience in conjunction with theoretical models that aim to provide the fullest explanation possible as to what it is to live with illness.

Todres, Galvin and Dahlberg’s [12] existential-phenomenological theory of lifeworld-led care explores how individuals live in relation to time, space, body, others and mood; a holistic outlook in which human beings understand the world around them. The lifeworld, as introduced by Husserl [13], and Heidegger [14], is a concept that represents the world as meaningful and relational, one that can be experienced and lived [15, 16]. Todres and Galvin [17] discussed six dimensions that represent constituents of the lifeworld, intertwined within one another, yet holding certain nuances of lived experience. The dimensions are temporality, spatiality, intersubjectivity, embodiment, identity and mood (see Table 1 for an explanation of the dimensions of the lifeworld). Arguably, many of the principles within pre-existing QoL domains complement the lifeworld-led care constructs. However, lifeworld-led theory allows for a more open, less-constrained view, which incorporates the shared and unique dimensions of individuals’ experiences that are central to their QoL: something which is particularly important when looking at a rare disease about which we know very little [12].

**Table 1:** Elements of the lifeworld adapted from Todres et al. [12]

Element of the lifeworld	Description
Temporality	Refers to time as it is humanly experienced, as a story through which we give order to our existence. One may have feelings of hope or opportunity. Others may feel sadness as opportunity is taken away from them
Embodiment	How humans live in meaningful ways; moving and living as this body. Meaning is given to our bodily existence. For example, our senses provide meaning to us, our body and the space it fills physically reacts and changes when faced with different experiences
Intersubjectivity	Meaning is given to relationships, society, culture and that we are in the world with others and the ways we relate to individuals who we meet in our worlds can be very important to our meaningful living
Identity	An ontological security of being able and feelings of ‘I can’. Or feelings of lack of ability and uselessness; feelings of being an object, trying to fit into impersonal systems
Spatiality	Meaning is given to places, objects and situations that we hold in significance in daily living. A sense of adventure may be felt or a sense of restriction. The interpretation of the spaces in which people live and the stimuli by which they are surrounded
Mood	Mood has been defined as shaping our one’s spatial, temporal, intersubjective horizons and is intimate to how we find ourselves. Mood leads individuals to categorise and prioritise experiences in certain ways. Understanding the influence of mood in relation to the elements of the lifeworld will help secure an understanding of the quality of lived experience

In order to understand the markers of QoL for people living with NPC, one must explore and understand the impact of living with illness on the lifeworld of a person. As Bunge [18] argued: “A better understanding of the quality of life calls for more intense theoretical and methodological work rather than an increase in the amount of social and environmental statistics… data without ideas are sterile when not misleading” (p. 65). Therefore, the current study sought to develop age-appropriate, disease-specific QoL scales based on the lived experiences of people living with NPC using Todres, Galvin and Dahlberg’s [12] lifeworld-led care theory. These scales would be developed for the assessment of individuals who have NPC to enable healthcare professionals to better engage with patient’s wellbeing and to provide a useful measure of outcome in clinical and drug trials [19].

## Methods

NHS ethical approval was granted for this study to enable recruitment from NHS clinics and the NPUK charity (ethics number: 15/WM/0093). Signed informed consent was obtained from all participants. Due to the rarity of the disease, individual participant demographic information has been hidden due to the possibility of identification, but the mean and standard deviation of the ages of the participants has been provided.

### Scale development

The process followed gold standard scale development guidelines [20, 21]. A qualitative approach was chosen which aimed to conceptualise ‘quality of life’ from the subjective experiences of people living with NPC, with an exploratory analysis routed in phenomenology. This study interpreted ‘quality’ as being that which helps or prevents people from living an authentic life. Any disruptions to a person’s lifeworld were seen as a key avenue of exploration in how living with NPC may impact upon a person’s QoL. Used as an approach to research, phenomenology provides a way of exploring phenomena holistically as they are experienced in the world. The phenomenon in this study is living with NPC as experienced by people with a diagnosis and their parents or carers. This approach allowed for a deeper understanding of the existential meanings of living with NPC. In conjunction with the use of phenomenology, lifeworld-led theory was drawn upon (see, for example of a similar analysis [22]). Using the elements of the lifeworld deepened the analysis, producing the best possible explanations as to the effects of NPC on all aspects of QoL.

#### Participants

Fourteen semi-structured interviews were conducted with people diagnosed with NPC and/or parents or carers of people living with NPC (see Table 2 for participant information). Eight parents/carers of children were interviewed and 6 parents/carers of adults. When possible, children or adults with NPC also took part in the interview. One interview took place with an adult with NPC without their parent/carer present. All participants were recruited through the Niemann-Pick UK charity (NPUK), a charity that provides practical support, advice and information for people affected by NPC. Participants were recruited purposively to ensure the sample consisted of a similar ratio of males to females and participants were from across the age range. All were UK residents.

**Table 2:** Interviewed participant characteristics

Participant number	Gender of person with NPC	Interviewees	Type of interview
001	Female	Father	Face to face
002	Female	Patient and husband	Face to face
003	Male	Mother	Face to face
004	Male	Father	Face to face
005	Male	Mother	Face to face
006	Male	Mother	Telephone
007	Female	Mother	Telephone
008	Female	Mother	Telephone
009	Female	Mother	Telephone
010	Male	Patient and mother	Face to face
011	Male	Patient and mother	Skype
012	Male	Patient and father	Face to face
013	Male	Mother	Face to face
014	Female	Patient	Face to face

#### Data collection

An advertisement for the study was published on the NPUK Charity’s social media pages and interested participants contacted the researcher. Written consent was taken from people with NPC who were able to take part in the interview themselves and from the person with NPC’s parents/carers prior to interview. The interview schedule was developed in consultation with parents of children with NPC, and clinicians who work with patients with NPC and with NPUK. Participants were asked to talk about their experiences of living with NPC. Open-ended questions focused on the NPC story starting from diagnosis, moving to how life had changed since receiving this diagnosis. Some examples of these questions are ‘Can you tell me about the diagnosis process and how you felt during it?’ ‘Can you tell me how things are on a daily basis?’ ‘Can you tell me about how living with NPC has impacted upon your life?’ For parents or carers who were interviewed without the person with NPC being present, the researcher asked them to put themselves ‘in the shoes of the person they were caring for’. The schedule was not prescriptive or inclusive but acted as a guide in the interview in order to ensure the aims of the study were met. Interviews were recorded using a Dictaphone and transcribed verbatim.

#### Qualitative analysis of interviews

In order to ensure the items were age appropriate, data were analysed in two age groups, children with NPC 0–17 years and adults with NPC 18 years and over. The interview data were analysed initially using interpretative phenomenological analysis (IPA) [23]. IPA is a phenomenological method, but with a focus on hermeneutics which enables one to examine the subjective experience of living with NPC as well as participants’ own sense-making of that experience [23, 24]. IPA usually focuses on first-hand accounts, but in this study we have extended this to incorporate the biographic account of parents’/carers’ meaning-making of the experiences of the person they are caring for. This enabled us to further explore and understand the phenomenon of living with NPC. Transcripts were re-read several times, enabling the researcher to become familiar with the data and to become attuned to the participants’ experiences. Each transcript was annotated in depth, focusing on semantic content and striking language used by the participant, which may be related to the impact of NPC on their QoL. Themes were generated, which focused on the interrelation of patterns in the data by mapping connections between the exploratory notes and the data. Todres, Galvin and Dahlberg’s [12] lifeworld-led theory was then used to further make sense of the inductive themes arising from the IPA analysis. This theory was adopted because of its commitment to describing and understanding the lifeworld in its wholeness, as a set of interrelated dimensions incorporating both wellbeing and suffering. This involved a cyclical dialogue between the themes generated alongside the elements of the lifeworld, thus embedding the inductive analysis within existential-phenomenological theory. Themes and subthemes were explored in more detail by going through each element of the lifeworld using the lens of living with NPC and the impact it may have on QoL. Discussions were held within the research team throughout all phases of analysis so we could be confident that the interpretation of the data produced was the best possible explanation.

#### Item generation

Due to the neurological effects of NPC on patients, after conducting interviews it was decided that it would be more appropriate to develop all the scales as proxy scales, rather than ones for direct use with the patient as very few people with NPC would be able to answer a QoL questionnaire [25]. From the cyclical process between the IPA analysis and the lifeworld-led theory, a bank of all possible items which were considered to have an effect on an individual’s QoL were noted. This involved the construction of a matrix of all reported themes and subthemes with descriptive summaries/phrases relevant to QoL set against the elements of the lifeworld. Themes and lifeworld elements were cross-compared to produce a list of all potential items that could be used to describe the QoL of someone with NPC. Variations of similarly focused items were worded differently and were kept on the list to establish which item was worded the most appropriately. Examples of items in relation to the lifeworld elements are reported in Table 3 for children and Table 4 for adults. These possible items were then discussed within a team of psychologists, one of which was an expert in scale development and validation, as well as stakeholders who work with NPC patients and families. Similar worded items were omitted as a result of this discussion.

**Table 3:** Matrix of themes, lifeworld elements and example items for the NPC quality-of-life scale for children

Theme	Element of the lifeworld	Notes	Items for a QoL scale for children with NPC
Learning to live with the clinical manifestations of NPC	Temporality Embodiment Mood	Present mobility compared to past. Recognition of the rare of deterioration. Extent to which NPC has manifested on their physical being and the implication this has on how they feel about themselves and their future The felt sense of bodily self-deteriorating. No longer able to do what they could before Adjustment to physical deterioration and the possibilities that equipment and support care staff bring	My child feels content in day to day life My child feels sad because of NPC My child looks forward to new experiences in the future My child feels comfortable with their body
Relationships as security and enablement	Intersubjectivity Embodiment Mood	A felt sense of belonging provided by family and friends giving a sense of peace Continuity of care and recognition of healthcare professionals and support staff offering a sense of familiarity.	Family time is important to my child My child feels supported by siblings/friends My child sometimes feels alone Being in company with others is important to my child My child feels similar to other girls and boys their age
The continuous journey of adaptation creating opportunity	Embodiment Identity Intersubjectivity Spatiality Mood	Adjustment and utilising equipment Making changes/amendments to increase or sustain freedom Feelings of loss identity and journeying towards an acceptance of their new sense of self	My child feels different to other children My child enjoys new social experiences and environments My child copes well when things change at home The home space helps my child feel at peace

**Table 4:** Matrix of themes, lifeworld elements and example items for the NPC quality-of-life scale for adults

Theme	Element of the Lifeworld	Notes	Items for a QoL scale for children with NPC
Variegated experiences of loss	Temporality Spatiality Intersubjectivity Identity	Past was full of potential with regards to life goals. The present now comprises of loss and the future is no longer looked towards with a sense of excitement. It is instead seen as hopeless Loss of self-esteem and independence. Reliance on others Loss of careers and progression, which affected how they saw themselves and their sense of identity	The person with NPC feels confident in who they are as a person Because of NPC they are not able to do the things they would like to do On the whole, they feel content They feel more content at home than in other settings
The experience of diminishing control	Temporality Spatiality Identity Embodiment	Pre NPC diagnosis, felt in control. Now learning to cope with new body that they no longer feel in control of Feelings of disconnect from society. Feelings of embarrassment at not being able to control body in society	The person with NPC feels in control of their present They feel in control of what their future looks like They feel they are the person they want to be They feel they want to hide away from the world
Longing for social meaning	Embodiment Intersubjectivity Temporality Identity	Comparison of what life was like pre-diagnosis Lack of hope for the future in finding friends/close relationships Struggle to relate to others—no shared experiences Feelings of isolation and loneliness	The person with NPC feels disconnected from society They feel that they do no relate to other people They feel that being with friends takes their mind off things that they find difficult They feel that they have a lot of things in common with their friends
Hiddenness, perception and identity	Temporality Intersubjectivity Embodiment Identity Mood Spatiality	Rare and varied disease; struggle to find others to share experiences with Shell of body—looks healthy but body lets them down. Feelings of embarrassment around the cognitive manifestations of NPC Limbo land: unsure what their identity is Separates disability from self, does not belong to them, imposter	The person with NPC feels that they are the person they want to be They feel that things in life are meaningful to them They feel motivated every day to achieve what they want to achieve

A mixture of closed questions and open-ended questions was developed for the child and adult scales. The open-ended questions were intended to garner a richer perspective on any additional needs of the person with NPC; these questions were optional. All closed items were written as statements with a 5-point Likert response scale from 1 (never) to 5 (always). The time period that people are asked to reflect over in order to answer the questions is over the course of 4 weeks. As the scales were developed as proxy versions, parents/carers were reminded in the instructions to answer the questions from the perspective of how they felt the one they were caring for would answer; this questionnaire was not assessing their experiences, but the experiences of the ones for whom they were caring. The final child prototype scale (NPCQLQ-C) consisted of 28 items; the adult prototype scale (NPCQLQ-A) consisted of 38 items.

### Item reduction and reliability testing

#### Data collection

Adverts for the study were promoted by NPUK on their social media pages, with the link to the questionnaires, which could be answered using Qualtrics, a secure online survey. The study was also advertised at the NPUK annual conference where paper copies of the questionnaire were available for parents/carers to complete. Paper copies were available on request. The majority (*n* = 26) of questionnaires were completed at the NPUK conferences, where LA and RK were present. Participants were asked to complete the relevant NPCQLQ scale alongside a demographic questionnaire on both the person with NPC and the parent/carer, to collect information including gender, age, occupation and ethnicity. They were also asked to complete an NPC severity questionnaire, with information such as when NPC was diagnosed, current symptoms and medication. Parents/carers were asked to rate what they felt to be the severity of the condition for the person for whom they were caring (from 0 to 9 with 9 being most severe). Both the demographic and disease severity questionnaires were developed by the research team.

#### Cognitive interviews

Cognitive interviews with six participants (split equally between parents of children with NPC and carers of adults with NPC) were conducted [21]. The objectives of cognitive interviewing are to determine the appropriateness and comprehension of the items included in the questionnaire, providing insight to the suitability of the questions asked [21]. This process involved LA and RK speaking to the participants, asking them to discuss each item, what it meant to them and whether they were able to answer it on behalf of the person with NPC for whom they were caring. They were also asked if the instructions and rating scales were clear. No changes were made to the instructions and rating scales based on the cognitive interviews. Feedback on items were used for item reduction alongside the statistical analyses (see Results section).

#### Reliability analysis

Initial item reduction was based on examination of inter-item correlations, floor and ceiling effects and number of missing data points for any item. Internal consistency was assessed by calculating Cronbach’s coefficient alpha for the total scale, to demonstrate the extent to which the items in the scales measure the same concept [26]. Although a usual part of scale development, an exploratory factor analysis was not planned due to the small numbers of participants completing the scales as a consequence of the rarity of the condition.

## Results

Twenty-three parents/carers completed the NPCQLQ-C (child age in years mean = 8.61; S.D. = 4.22). Twenty parents/carers completed the NPCQLQ-A (adult age in years = 33.4, S.D. = 12.2). The sample comprised of 18 males; 20 females with NPC. One child’s and 4 adult’s gender were not reported. Mean proxy-rated NPC severity rating was 4.26 (S.D. 2.89) for children and for 4.45 (S.D. 2.23) for adults.

### Item reduction and reliability of the NPCQLQ-C—QoL scale for children

Floor and ceiling effects were checked and no items were removed. Inter-item correlations revealed nine items highly correlated with each other; 5 of these were removed. Removal decisions were made in line with the results of the cognitive interviews; participants felt that some items were too similar, for example: *“My child looks forward to new experiences in the future”* and *“My child is excited by the prospect of taking part in special occasions”*; for other items, some felt that they were not relevant, such as *“My child feels at peace with themselves”*.

Item-total correlations were run and eight items were removed because they did not correlate with the total score (correlations of 0.1 or 0.2) or because they had a negative item-total correlation. Upon reflection, these items could be interpreted differently by each participant, thus did not provide a direct relationship between a high score measuring high QoL. Cronbach’s *α* of the remaining 15 items was 0.925. A full list of items is reported in Table 5. These items were summed and correlated with the item asking respondents to rate the severity of NPC in the child they were caring for. A higher severity rating significantly correlated with poorer QoL with a large effect size (*r* = − 0.094).

**Table 5:** Final items included in the NPCQLQ-C

	Item		Item
1	Because of NPC, my child’s mobility has been affected	9	My child enjoys spending time with their friends
2	Because of NPC, my child struggles to hold things in his or her hand	10	Family routines make my child feel secure
3	My child enjoys playing with their toys or equivalent	11	I think my child feels like they cannot do things other children their age can do because of NPC
4	My child feels content about their physical health	12	I think my child feels like their relationships with their friends have changed because of NPC
5	My child feels comfortable with their body	13	Changes to our home environment have made day to day living easier for my child
6	My child is excited by the prospect of taking part in special occasions	14	My child appears to be sad
7	My child experiences times of frustration regarding their symptoms of NPC	15	My child copes well when things change at home
8	My child generally enjoys daily life		

### Item reduction and reliability of the NPCQLA-A—QoL scale for adults

Floor and ceiling effects were checked and no items were removed. Inter-item correlations revealed nine items highly correlated with each other and 5 of these were removed. Items were removed based on results from the cognitive interviews. Participants felt that some items were too similar, for example: *“They feel content in day to day life”* and *“On the whole they feel content”*; for other items, some felt that they were not relevant, for example: *“In general, they feel that their physical state does not allow them to do the things they want to do.*”

Item-total correlations were run on the remaining 33 items. Three items were removed because they did not correlate with the total score (correlations of 0.1 or 0.2) or they had a negative item-total correlation. Again upon reflection these items could be interpreted differently by each participant, therefore did not provide a direct relationship between high score and high QoL. Cronbach’s *α* of the remaining 30 items was 0.947, indicating excellent internal consistency. A full list of items is in Table 6. These items were summed and correlated with the item asking respondents to rate the severity of NPC in the person they were caring for. A higher severity rating significantly correlated with poorer QoL with a large effect size (*r* = − 0.080).

**Table 6:** Final items included in the NPCQLQ-A

	Item		Item
1	The person with NPC feels supported in daily life	16	They feel that others do not always accept them
2	They feel hopeful when they think about the future	17	They feel motivated every day to achieve what they want to achieve
3	They feel that NPC has affected their social interactions	18	They feel that their body does not allow them to do things that they want to
4	They feel that they do not relate to other people	19	They find is easy to make new friends
5	They feel isolated	20	The person with NPC feels content when they think about the past
6	They feel content with their body and how it works	21	They feel in control of the present
7	They feel uncomfortable with the busyness of the world	22	They enjoy being in busy environments
8	They feel that sometimes other people do not understand them	23	Because of NPC they are not able to do the things that they would like to do
9	They feel in control of their life	24	How people view them is important to them
10	They feel confident in who they are as a person	25	They sometimes like to hide away from the world
11	They feel that friendships have changed over time	26	They have good relationships with healthcare staff such as doctors and nurses
12	They sometimes feel that they would like to have closer relationships with others	27	In general, they feel well
13	They feel disconnected from society	28	The person with NPC often feels low
14	They enjoy being part of the local community	29	They feel overwhelmed by different situations
15	The person with NPC would describe themselves as being an independent person	30	On the whole, they feel at peace

## Discussion

Adopting a phenomenological approach to explore the experiences of living with illness allowed for a richer understanding of how QoL is impacted upon when living with NPC. Using the elements of the lifeworld enriched our understanding of participants’ subjective experiences which then ensured the items included in the scale represented participants’ worlds when living with NPC. The initial reliability tests demonstrate the excellent internal consistency of the items in the scales for children and adults living with NPC. This information could be used to direct healthcare to more appropriate areas and to provide information for patients, parents/carers and healthcare practitioners on the effectiveness of drug treatments and other health-related interventions for those living with NPC.

The NPCQLQ-C items focused on the embodied experience of living with NPC, both in terms of the ‘objective’, physical manifestation of NPC, and the subjective, in terms of children’s contentment with being in their body. Prognosis varies but people who display neurological symptoms in childhood have been shown to deteriorate faster compared to those who become symptomatic later on in life [27]. Items around whether children could play with their toys or whether NPC had affected their embodiment were shown to be important to a child’s QoL in that they could no longer take part in activities they could previously and the frustration this may arouse.

The NPCQLQ-A items focused more on the intersubjective sense of self and how this related to the temporal element of the life they once knew before their diagnosis with NPC, compared to the life they are now living. Many of the adults diagnosed with NPC had to leave employment and their social relationships had changed. For many, a change in identity was felt, and this led to spells of anxiety and depression due to the life they felt they had lost. Although many of the adults had better physical health than children with NPC, the impact on QoL could be greater, highlighting the complexity of the effects of this life-limiting condition on a person’s QoL and how this is not necessarily directly associated with the physical/objective body. Without explicitly exploring each element of the lifeworld, these nuances in people’s experiences may not have been identified.

Furthermore, drawing on the lifeworld theory means that both measures, the NPCQLQ-C and NPCQLQ-A are theoretically underpinned by an existential-phenomenological theory which transcends the individual. This also means that we have been able to go beyond concepts of QoL used in more ‘traditional’ measures. The intention of such a design is to prioritise the concerns of the individuals involved by first exploring the nature of their experience and moreover, to examine the meanings those people attribute to their experiences. This is especially valuable when working with such a rare condition because it then means that these meaningful experiences, rather than preconceived notions from other QoL scales, are used as the starting point for a measurement that can then be used in clinical practice and research.

Using this method may help people who are answering the questionnaire to be reflective, which in turn allows for concrete experiences to come to the fore rather than relying on what might be construed as more abstract concepts of QoL. This too aids healthcare professionals and researchers in understanding more about the lifeworld of their patients and the impact of living with NPC on their everyday lives. At the heart of this lifeworld-led approach is a commitment to humanising healthcare, i.e. ensuring that we treat the whole person rather than simply the condition. This study has highlighted the significance of how living with NPC may affect the patient’s sense of self and bodily identity rather than purely focusing on the physiological impact. These scales could be used as a guide in advancing healthcare and policies to better engage with the illness experience in a way that humanises care [28]. This approach has aimed to be sensitive to what really matters to people living with NPC. The next part of the process will be to assess the validity of both the NPCQLQ-C and NPCQLQ-A in a comparative analysis with other validated QoL scales.

### Implications for practice

It is important for healthcare professionals to have a more in-depth understanding of the impact of living with NPC on a patient’s QoL. Understanding patients’ and families’ experiences of living with NPC is an under-researched area which could be revolutionary in shaping healthcare professionals’ approaches to treatment for NPC and the impact of those treatments on a person’s QoL. Both the NPCQLQ-C and the NPCQLQ-A provide a robust ‘measurement’ or representation of the more subjective, experiential impact of NPC on an individual’s QoL. Working in a way that prioritises subjective experience and offers a more humanising approach to care would enable services to deliver support that focuses on meaningful person-centred care.

### Limitations

A key concern for this work is the small numbers of participants that completed the QoL questionnaires. However, with such a rare condition this is not surprising. For both the NPCQLQ-C and the NPCQLQ-A, nearly 50% of the diagnosed population in the UK completed a questionnaire, which proportionally offers a good representation of the diagnosed population. In addition, condition-specific scales have been criticised for their narrowness in focus, because they neglect to measure more general outcome variables [29]. However, it is important, especially in rare diseases, to understand the issues that are specific to people living with that condition and very little is known about NPC in this respect. Condition-specific scales measuring QoL are more useful in detecting changes in a condition over time compared to a generic scale [30]. This allows for the domains of QoL that are important to the population in question to be described and quantified [19].

## Conclusion

The NPCQLQ-C and the NPCQLQ-A were developed to capture the experiential, real life impact of NPC on patients’ daily lives for the purposes of both clinical practice and research. For example, they may be used in the assessment of QoL during the development, evaluation and implementation of new treatments and other interventions in clinical trials. These are the first disease-specific tools to measure QoL in this population and will enable healthcare professionals and researchers to have a better understanding of the impact of living with this rare disease on a patient’s daily life. The initial reliability analysis of both scales shows excellent internal consistency and they are highly correlated with disease severity, which suggests there is good reason to test and evaluate their use in clinical practice. We argue that using the scales would give voice to patients and would promote more dialogue between patients, parents/carers and healthcare professionals that is grounded in lived experience thereby delivering meaningful person-centred care.
